# Efficient Generation of Multipotent Mesenchymal Stem Cells from Umbilical Cord Blood in Stroma-Free Liquid Culture

**DOI:** 10.1371/journal.pone.0015689

**Published:** 2010-12-30

**Authors:** Rowayda Peters, Monika J. Wolf, Maries van den Broek, Mario Nuvolone, Stefanie Dannenmann, Bruno Stieger, Reto Rapold, Daniel Konrad, Arnold Rubin, Joseph R. Bertino, Adriano Aguzzi, Mathias Heikenwalder, Alexander K. Knuth

**Affiliations:** 1 Clinic of Oncology, University Hospital of Zurich, Zurich, Switzerland; 2 Department of Pathology, Institute of Neuropathology, University Hospital of Zurich, Zurich, Switzerland; 3 Department of Medicine, Institutes of Clinical Pharmacology and Toxicology, University Hospital of Zurich, Zurich, Switzerland; 4 Division of Diabetology und Endocrinology, University Children's Hospital, Zurich, Switzerland; 5 Department of Medicine, The Cancer Institute of New Jersey, Robert Wood Johnson Medical School, University of Medicine and Dentistry, New Brunswick, New Jersey, United States of America; 6 Department of Internal Medicine, Amyloid Center, Fondazione IRCCS Policlinico San Matteo, University of Pavia, Pavia, Italy; 7 Institute of Virology, Technische Universität München/Helmholtz Zentrum München, München, Germany; Massachusetts General Hospital, United States of America

## Abstract

**Background:**

Haematopoiesis is sustained by haematopoietic (HSC) and mesenchymal stem cells (MSC). HSC are the precursors for blood cells, whereas marrow, stroma, bone, cartilage, muscle and connective tissues derive from MSC. The generation of MSC from umbilical cord blood (UCB) is possible, but with low and unpredictable success. Here we describe a novel, robust stroma-free dual cell culture system for long-term expansion of primitive UCB-derived MSC.

**Methods and Findings:**

UCB-derived mononuclear cells (MNC) or selected CD34^+^ cells were grown in liquid culture in the presence of serum and cytokines. Out of 32 different culture conditions that have been tested for the efficient expansion of HSC, we identified one condition (DMEM, pooled human AB serum, Flt-3 ligand, SCF, MGDF and IL-6; further denoted as D7) which, besides supporting HSC expansion, successfully enabled long-term expansion of stromal/MSC from 8 out of 8 UCB units (5 MNC-derived and 3 CD34^+^ selected cells). Expanded MSC displayed a fibroblast-like morphology, expressed several stromal/MSC-related antigens (CD105, CD73, CD29, CD44, CD133 and Nestin) but were negative for haematopoietic cell markers (CD45, CD34 and CD14). MSC stemness phenotype and their differentiation capacity *in vitro* before and after high dilution were preserved throughout long-term culture. Even at passage 24 cells remained Nestin^+^, CD133^+^ and >95% were positive for CD105, CD73, CD29 and CD44 with the capacity to differentiate into mesodermal lineages. Similarly we show that UCB derived MSC express pluripotency stem cell markers despite differences in cell confluency and culture passages.

Further, we generated MSC from peripheral blood (PB) MNC of 8 healthy volunteers. In all cases, the resulting MSC expressed MSC-related antigens and showed the capacity to form CFU-F colonies.

**Conclusions:**

This novel stroma-free liquid culture overcomes the existing limitation in obtaining MSC from UCB and PB enabling so far unmet therapeutic applications, which might substantially affect clinical practice.

## Introduction

In recent years, mesenchymal stem cells (MSC) received considerable attention as a potential source of cell-based therapies and as a cell type that supports the engraftment of haematopoietic stem cells (HSC) [1], [2]. The usual source of MSC is the bone marrow (BM), which is not easy to obtain from healthy donors as well as umbilical cord blood (UCB). The advantages of UCB as the source of MSC are the availability of units [3], [4] and the primitive nature of UCB-derived MSC [5], [6], [7].

BM- and UCB-derived MSC are presumably highly similar precursors as they share the following features: (*i*) capacity of self-renewal [8], (*ii*) multipotency, allowing *in vitro* differentiation into mesenchymal tissues (bone, cartilage, tendon, muscle, adipose tissue, stroma) and possibly non-mesenchymal tissues (neuronal, endothelial and hepatic) [9], [10], [11], (*iii*) formation of colonies of fibroblastic cells (CFU-F) [12], (*iv*) expression of MSC markers (CD29, CD44, CD73, CD105) and lack of haematopoietic markers (CD14, CD34, CD45) [4], [13] and (*v*) migration to inflammatory sites, stimulation of proliferation/differentiation of resident progenitor cells and promotion of recovery of injured cells through growth factor secretion and matrix remodeling [14], [15], [16].

Although the frequency of MSC referred here as undifferentiated cells is much higher in BM (0.001–0.1%) than in UCB (0.00003%) and some reports have even doubted the presence of MSC in UCB [17], [10], [4], [18], [19], UCB-derived MSC have a better potential to expand and can give rise to up to 10^15^ cells [17], [4]. However, the scarcity of MSC in UCB and the lack of a robust protocol to reproducibly expand MSC from UCB units have hampered clinical applications [17], [4], [20], [19]. It can not be excluded that the low numbers of MSC in cord blood actually derive from placental MSC that were released into cord blood due to mechanical stress during UCB isolation procedure. Several studies reported that MSC can be isolated and established from only 20–63% of the cord blood units [3], [8], [21], questioning the feasibility of MSC isolation and cultivation from UCB.

Here, we describe a novel, simple and reproducible method, which is based on stroma-free liquid culture, to expand substantial numbers of multipotent MSC from only a small number of UCB-derived mononuclear cells (MNC). This method allows an extensive expansion of non-adherent HSC plus a marked increase in adherent MSC. MSC produced *in vitro* by this novel culture method maintain their stem cell properties of self-renewal and multi-lineage differentiation for a long-time (up to passage 24), even following cryopreservation.

## Methods

### Ethics Statement

All experimental work presented in this study has been approved by the local institutional review board.

### Umbilical cord blood sample collection and cell processing

UCB from full-term deliveries was obtained from the Department of Gynaecology at the University Hospital of Zurich with the approval of the local ethical committee (Beschlussmitteilung der Ethikkommission, UniversitätsSpital Zürich, and StV 30/2006). A written informed consent was obtained and the blood was processed within 24 h. Briefly, UCB was diluted with an equal volume of PBS layered onto Lymphoprep™ (Axis-Shield, UK) and centrifuged for 30 min at room temperature at 1′800 rpm. Mononuclear cells (MNC) were collected and washed twice with PBS. Aliquots containing 1.0×10^7^ cells were cryopreserved in FBS and 10% DMSO at a fixed cooling rate (1°C/min) and stored at −80°C.

### Cytokines

Recombinant human stem cell factor (SCF), interleukin 6 (IL-6), FMS-like tyrosine kinase 3 (Flt-3) ligand, epidermal growth factor (EGF), basic fibroblast growth factor (FGF-β), hepatocyte growth factor (HGF) and Oncostatin M (OSM) were purchased from R&D Systems (Europe). Megakaryocyte growth and development factor (MGDF) was a gift from Kirin (Gunma, Japan).

### Cell selection

UCB CD34^+^ cells were positively selected using autoMACS CD34^+^ magnetic beads (Miltenyi Biotec, Bergisch Gladbach, Germany). The separation technique was performed according to the manufacturer's instructions. The purity of CD34^+^ cells selected from 3 UCB units ranged between 90–92.7% as determined by flow cytometry.

### Stroma-free long-term cultures

Cryopreserved MNC were thawed and cultured for the expansion of HSC in Dulbecco's Modified Eagle's Medium (DMEM, Gibco, Europe) in the presence of 10% pooled human AB serum (Cat. No. 100–512, SLI, UK) in 24-well plates. For expansion, freshly thawed UCB-derived MNC were seeded at a concentration of 1–3×10^5^/ml. SCF and Flt-3 were used at 25 ng/ml, MGDF at 10 ng/ml and IL-6 at 20 ng/ml. Flt-3, SCF and MGDF were added on day 0 to initiate the cultures and IL-6 was added on day 7-10 (Table 1, Table S1). UCB cultures were grown at 37°C in humidified 5% CO_2_ in air. The cells were fed with fresh media every 3 days. For selected CD34^+^ cells, 50′000–250′000 cells/ml were obtained from 3 UCB units and were cultured in condition D7 as described above.

**Table 1:** Thirty two different culture conditions tested for the expansion of human umbilical cord blood (UCB) derived hematopoietic precursor cells.

Cytokine Cocktail (CC)	Plasma* 2%	Plasma* 8%	Serum* 10%	FCS 10%	Culture Type
CC1	RPMI	RPMI	RPMI	RPMI	A1-A4
CC1	DMEM	DMEM	DMEM	DMEM	A5-A8
CC2	RPMI	RPMI	RPMI	RPMI	B1-B4
CC2	DMEM	DMEM	DMEM •	DMEM	B5-B8
CC3	RPMI	RPMI	RPMI	RPMI	C1-C4
CC3	DMEM	DMEM	DMEM •	DMEM	C5-C8
CC4	RPMI	RPMI	RPMI	RPMI •	D1-D4
CC4	DMEM	DMEM	DMEM ▴	DMEM	D5-D8

*Plasma and serum are derived from human blood group AB. FCS: fetal calf serum.

CC1  =  SCF (10 ng/ml) + IL-3 + IL-6 (100 ng/ml) + IL-1β (3 ng/ml) + EPO (1 U/ml), [25].

CC2  =  Flt-3 (50 ng/ml) + MGDF (10 ng/ml), [26].

CC3  =  Flt-3 (50 ng/ml) + MGDF (10 ng/ml) + IL-6 (10 ng/ml) + SCF (50 ng/ml), [27].

CC4  =  Flt-3 (25 ng/ml) + SCF (25 ng/ml) + MGDF (10 ng/ml) + IL-6 (20 ng/ml), modification of [27].

▴D7 (CC4 + DMEM + 10% serum) maintained long-term expansion of UCB HSC over 7 months.

•B7 (CC2 + DMEM + 10% serum), C7 (CC3 + DMEM + 10% serum) and D4 (CC4 + RPMI +10% FCS) maintained short- term expansion for 6 weeks.

### Culture of UCB-derived MSC during HSC expansion

On day 14, expanded UCB-derived non-adherent cells, referred to as HSC, were removed from the 24-well plates. The remaining adherent cells, referred to as stromal/MSC, were washed with PBS and thereafter either enriched in culture medium containing 10% fetal bovine serum (FBS; Sera Laboratories International, UK) in DMEM (Sigma-Aldrich Company Ltd, UK), supplemented with 2 mmol/l L-glutamine, 50 IU/ml penicillin, 50 µg/ml streptomycin (Gibco BRLR Life Technologies Ltd, UK), 10 ng/ml FGF-β and 20 ng/ml EGF or in MesenCult and added supplements (StemCell Technologies, Europe) in the presence of 5 ng/ml FGF-β (Table 1, Table S1, Fig. S1). The cultures were maintained at 37°C in a humidified atmosphere containing 5% CO_2_. The medium was changed twice weekly. When adherent cells increased in number after 2 weeks, they were passaged, counted and transferred to a 25 cm^2^ flask for further expansion. The procedure until passage (P) 1 of stromal/MSC takes 28 days.

### CFU-F assay

The colony forming unit fibroblast assay (CFU-F) was first performed after the first passage at day 28 and also later during expansion. MSC (10′000/25 cm^2^ tissue flask) were either cultured in 10% FBS in DMEM supplemented with 2 mmol/l L-glutamine, 50 IU/ml penicillin, 50 µg/ml streptomycin and 10 ng/ml FGF-β and 20 ng/ml EGF or in MesenCult and added supplements in the presence of 5 ng/ml FGF-β. CFU-F count was determined at day 14 and a coherent group of ≥10 cells was counted as one colony. After P10, CFU-F count was determined at day 8 instead of day 11 to avoid the overlap between colonies due to a rapid increase in cell division.

### Differentiation of MSC

Cultured cells were harvested with 0.5% trypsin-EDTA (Invitrogen AG, Switzerland). Cells at passages 4 or 5, or when the expression of the haematopoietic markers CD45, CD34 and CD14 was ≤1.5%, were seeded in 25 cm^2^ tissue culture flasks. MSC were stimulated to differentiate into adipocytes (fat), osteoblasts (bone) and hepatocytes (liver) as follows. At 80% confluence, the cells were treated in adipogenic differentiation medium (Stem Cell Technologies, Europe). The medium was changed twice weekly for 3 weeks. For differentiation into osteoblasts, 3×10^4^ cells were incubated in MACSR NH OsteoDiff medium, according to the manufacturer's instructions (Miltenyi Biotec, Germany). The medium was changed every 3 days. To induce hepatogenic differentiation, cells were treated at 80% confluence with differentiation medium, containing DMEM supplemented with 20 ng/ml HGF, 0.5 mM dexamethasone, 50 mg/ml ITS premix, 2 mmol/l L-glutamine and 50 IU/ml penicillin and 50 µg/ml streptomycin for 14 days followed by maturation thereafter. Maturation medium contained the same reagents as differentiation medium except HGF, which was replaced with OSM (20 ng/ml) (10). Medium changes were carried out twice weekly and differentiation into hepatogenic, adipogenic and osteogenic cells was assessed by flow cytometry and real-time PCR at day 10 and 21 and by immunohistochemistry at day 28.

### Isolation of adipocytes from human fat tissue

One g of tissue (obtained from a healthy volunteer following the approval of the local ethical committee [Beschlussmitteilung der Ethikkommission, UniversitätsSpital Zürich, EK 647]) was cut into small pieces. The tissue was digested in 10 ml Krebs-Ringer phosphate-HEPES buffer containing 4% fatty acid-free BSA and 1 mg/ml of type I collagenase (Worthington). Digestion was performed in a gyratory water bath at 180 rpm for 45–50 min at 37°C. At the end of the incubation, contents of the vial were filtered through a 250 µm nylon filter (Nitex) into a 50 ml polypropylene conical tube. The filter was rinsed with 10 ml Krebs-Ringer phosphate-HEPES buffer containing 1% BSA (KRB 1%). The filtrate was centrifuged at room temperature at 1′000 rpm for 2 min to float the white adipocytes. Adipocytes were transferred into a 50 ml tube and washed by addition of 10 ml KRB 1% BSA followed by centrifugation for 2 min at 1′000 rpm. Fluid and possible erythrocytes in the pellet were aspirated, the same amount of buffer was added and the tube was slightly shaken. Washing was repeated three times and cells were suspended in 1.5 ml.

### Phenotypic analysis

#### (I) Flow cytometry

To phenotypically characterize MSC, cells were surface stained with monoclonal antibodies specific for CD45, CD34, CD14, CD73, CD105, CD44, CD29, CD133, (HLA)-ABC (MHC class I cell surface receptor), HLA-DR (MHC class II cell surface receptor) and Nestin. Additionally, cells were stained intracellularly for cytokeratin18 (CK18), PPARγ and osteopontin (OPN) after fixation and permeabilization (Perm Buffer II, BD Phosflow, Europe), according to the manufacturer's instructions. All stainings were performed at 4°C for 30 min. The following cell lines were used as positive controls for differentiated cells: Huh7 for hepatocytes, CRL-11372 for osteoblasts and human adipose tissue for adipocytes and undifferentiated MSC were used as negative control. All antibodies were obtained from BD Biosciences (Europe) except CD133 (Miltenyi Biotec GmbH, Germany), and PPARγ and OPN (Santa Cruz Biotechnology, Germany). Appropriate isotype-matched, nonreactive fluorochrome-conjugated antibodies were used as controls. Cells were stained and analyzed as described previously [22], [23], [24] using a FACS Calibur flow cytometer (BD Biosciences) and data were analyzed using CellQuest Pro software.

#### (II) Real-time PCR

Total RNA was isolated from 2×10^6^ MSC-derived cells using RNeasy kit (Qiagen), according to the manufacturer's instructions. The quantity and quality of the RNA was determined spectroscopically using a nanodrop (Thermo Scientific). Purified RNA was DNase treated and subsequently reversely transcribed into cDNA using Quantitect Reverse Transcription Kit (Qiagen) according to the manufacturer's protocol.

For mRNA expression analysis real-time PCR was performed using Fast Start SYBR Green Master Rox (Roche). Primers were custom made by Microsynth (Switzerland). The following primers were used for the evaluation of human cytokeratin 14, cytokeratin 18, albumin, osteopontin, alkaline phosphatase, igfbp2, pparγ2, lpl, nestin, oct3/4, nanog, sox2 and rex1 mRNA expression:

ck14 fwd: 5′-GGG CGG CCT GTC TGT CTC-3′; ck14 rev: 5′-AGG CGG TCA TTG AGG TTC TG-3′; ck18 fwd: 5′-CCC GCT ACG CCC TAC AGA-3′; ck18 rev: 5′-GCG GGT GGT GGT CTT TTG-3′; albumin fwd: 5′-GCT GCC ATG GAG ATC TGC TTG A-3′; albumin rev: 5′- GCA AGT CAG CAG GCA TCT CAT C-3′; osteopontin-fwd: 5′-CTC CAT TGA CTC GAA CGA CTC-3′; osteopontin-rev: 5′-CAG GTC TGC GAA ACT TCT TAG AT-3′, alpl-fwd: 5′-CAC CCA CGT CGA TTG CAT CT-3′; alpl-rev: 5′-TAG CCA CGT TGG TGT TGA GC-3′; pparγ2-fwd: 5′-CCT ATT GAC CCA GAA AGC GAT T-3′; pparγ2-rev: 5′-CAT TAC GGA GAG ATC CAC GGA-3′; igfbp2-fwd: 5′-GAC AAT GGC GAT GAC CAC TCA-3′; igfbp2-rev: 5′-GCT CCT TCA TAC CCG ACT TGA-3′; lpl-fwd: 5′-TCA TTC CCG GAG TAG CAG AGT-3′; lpl-rev: 5′-GGC CAC AAG TTT TGG CAC C-3′; nestin-fwd: 5′-GGC GCA CCT CAA GAT GTC C-3′; nestin-rev: 5′-CTT GGG GTC CTG AAA GCT G-3′, oct3/4-fwd: 5′-CCC TCG TGC AGG CCC GAA AG-3′; oct3/4-rev: 5′-AAG CTG CTG GGC GAT GTG GC-3′; nanog-fwd: 5′-CCT TGG CTG CCG TCT CTG GCT-3′; nanog-rev: 5′-AGC AAA GCC TCC CAA TCC CAA-3′; sox2-fwd: 5′-AGG GGG AAA GTA GTT TGC TGC CT-3′; sox2-rev: 5′-TGC CGC CGC CGA TGA TTG TT-3′; rex1-fwd: 5′-AGG CCA GTC CAG AAT ACC AG-3′; rex1-rev: 5′-TAG GTA TCC GTC AGG GAA GC-3′.

Real-time PCR was performed on an ABI PRISM 7700 Sequence Detection System (Applied Biosystems). The mRNA expression level for each gene was normalized against 18S rRNA (Applied Biosystems). Data was generated and analyzed using SDS 2.3 and RQ manager 1.2 software.

#### (III) Immunohistochemistry

Differentiation to adipocytes was recognized by the accumulation of lipid-containing vacuoles stained with Oil red O (Miltenyi Biotec GmbH). Differentiation into osteoblasts is characterized by alkaline phosphatase activity, which was demonstrated using Fast BCIP/NBT as a substrate (Sigma, Europe).

#### (IV) Immunocytochemistry

Differentiation into hepatocyte-like cells was confirmed by staining for intracellular albumin. For this purpose, cytospin slides were prepared from the MSC cultures, were fixed for 30 min in cold methanol at 4°C and were incubated for one hr with FITC-conjugated polyclonal rabbit antibody against human albumin. Before and after incubation, slides were washed with PBS. Cytospins were prepared from expanded stromal/MSC from one UCB at P7, P13 and P24. These were stained with CD133, Nestin, CD45, and mouse isotype control antibodies at the routine pathology laboratory. In addition, cytospins were prepared from expanded UCB MNC at week 6 in stroma-free liquid culture and from control cells obtained from BD Biosciences Europe, (Cat. No. 340991) and were stained with CD34 monoclonal antibody. Positive staining was demonstrated with peroxidase reaction and the staining was repeated twice.

#### (V) Transmission electron microscopy

Adherent stromal/MSC cultures were fixed with 3% glutaraldehyde in PBS for 40 min. Subsequently, the culture was washed three times with PBS prior to post fixation with 2% OsO_4_ in PBS for 30 min. Cells were rinsed three times with nanopure water and dehydrated in a sequence of increasing ethanol/water mixtures for 30 min each, 70%, 96%, 100% and 100% water-free ethanol (twice). Embedding was carried out with 33% and 50% Epon in ethanol for 2 hrs each, 75% Epon in ethanol over night and 100% Epon for 2 hrs before polymerization at 60°C for 48 hrs. Thin sections were stained with aqueous uranyl acetate 2% and Reynolds lead citrate and imaged in a Phillips CM 12 transmission electron microscope (FEI, Eindhoven, Netherlands) using a Gatan CCD camera (1k×1k) and digital micrograph acquisition software (Gatan GmbH, Munich, Germany).

#### (VI) Peripheral blood (PB) MNC preparation and stroma-free liquid culture

PB-MNC were obtained from 8 healthy volunteers. The expansion procedure was carried out as described for UCB (see above). Freshly thawed PB-derived MNC were seeded in culture condition D7 at a concentration of 1×10^6^/ml. The stromal/adherent cells generated in D7 were either enriched in culture medium containing DMEM, supplemented with 2 mmol/l L-glutamine, 50 IU/ml penicillin, 50 µg/ml streptomycin + different concentrations of FBS (10%, 15% and 20%) or + 10% pooled human AB serum, in the presence of 5 or 10 ng/ml FGF-β and 5, 10 or 20 ng/ml EGF or in MesenCult + supplements ±5 and 10 ng/ml FGF-β. The cultures were maintained at 37°C in a humidified atmosphere containing 5% CO_2_.

## Results

### Generation of UCB-derived MSC during HSC expansion

**Figure 1:**
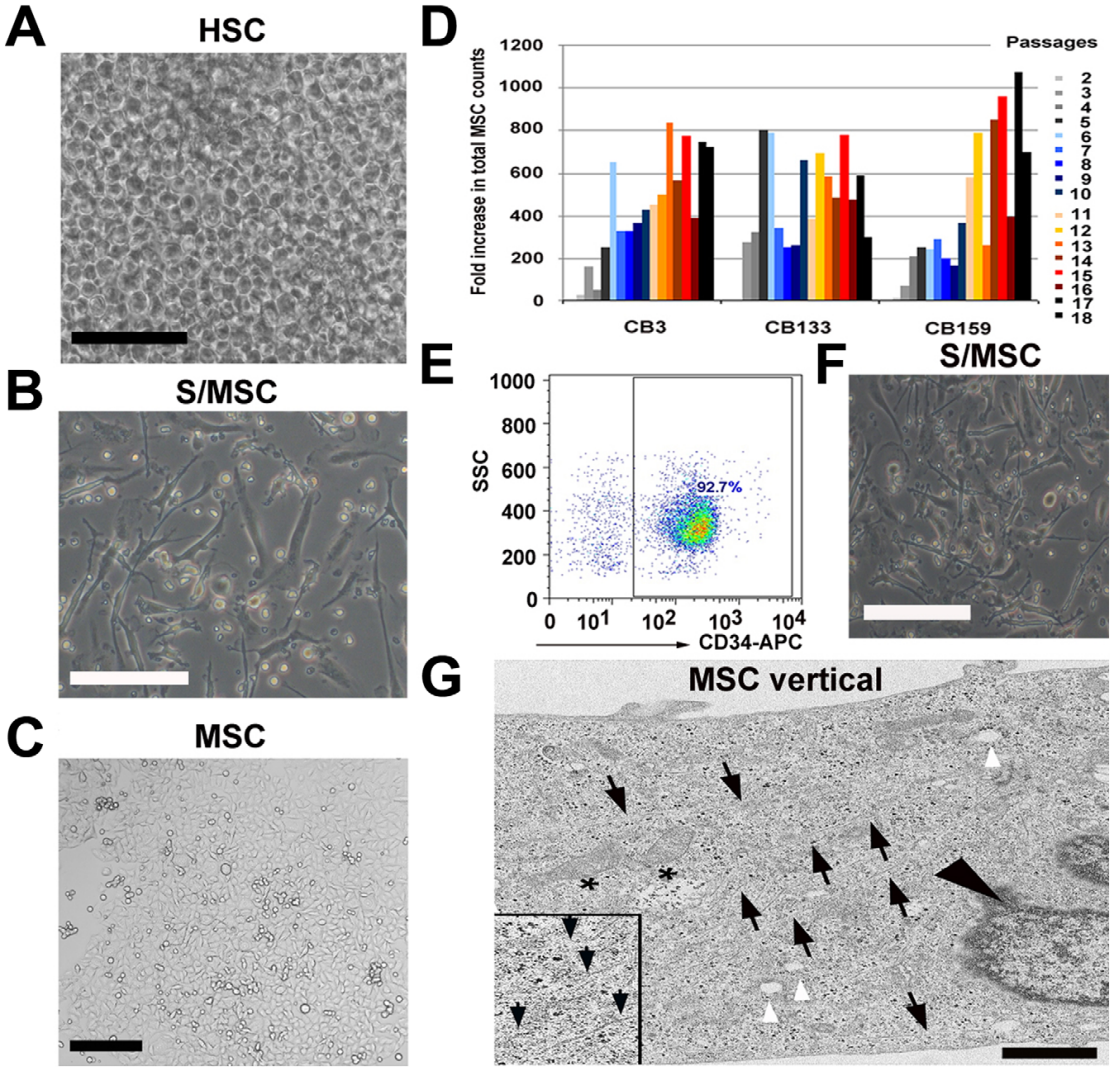
Umbilical cord blood stromal/adherent MSC generated during the expansion of haematopoietic stem cells in stroma-free liquid culture. (A) Confluent growth of HSC from cultured UCB-derived MNC after 14 days expansion under stroma-free culture condition D7. Viable cell count was determined weekly (scale bar: 150 µm). (B) Adherent stromal cells after removal of non-adherent haematopietic cells and further cultivation in MesenCult with added supplements and 5 ng/ml FGF-β for an additional 14 days (scale bar: 150 µm). (C) Morphology of expanded UCB-derived MSC after 165 days/P11 in culture. After passage 1, cells were transferred from 24-well plate to 25 cm^2^ culture flasks and plated at a density of 80–400 cells/cm^2^ in MesenCult with added supplements and 5 ng/ml FGF-β. At 80% confluency, the cells were passaged again (scale bar: 100 µm). (D) Total MSC counts of 3 independent human cord blood units (CB3, CB133, CB159) performed from P2 through P18. Different colors depict different passages (2–18). Fold increase was measured by dividing the total MSC count by the starting cell number, which was 10′000 cells/25 cm^2^ tissue flask. (E) Percentage CD34^+^ selected cells using autoMACS CD34^+^ magnetic beads as determined by flow cytometry. (F) Adherent stromal cells generated from CD34^+^ selected cells expanded in D7 culture condition for 14 days. Non-adherent haematopoietic cells were removed and adherent cells were cultivated in MesenCult with added supplements and 5 ng/ml FGF-β for an additional 14 days (scale bar: 150 µm). (G) Electron microscopy (EM; vertical section) of a stromal/MSC at 198 days/P14 in culture. White arrowheads point to vacuoles and black arrowhead depicts the nucleus. Asterisks show irregular mitochondria and black arrows point to various filaments (inset, black arrows) characteristic for stromal cells. Black dots within the cytoplasm represent ribosomes (scale bar: 1 µm).

Unexpectedly, the stroma-free liquid culture, which was initiated to establish culture conditions to expand HSC from freshly thawed MNC, also generated fibroblast-like cells, which we tentatively term UCB-derived MSC (UCB-MSC). Isolation of MSC rested on their classic adhesion on tissue culture plastic. We systematically tested 32 different culture conditions to expand HSC from various independent UCB units. We used various combinations of growth factors, cytokine cocktails [25], [26], [27], protein sources (FBS, pooled human AB serum or plasma) at different concentrations and basic culture media (Table 1, Fig. S1). Only 4 out of 32 conditions supported the expansion of HSC and 3 of those 4 conditions maintained HSC expansion only temporarily (up to 6 weeks), whereas only condition D7 (Table 1, Fig. 1A) allowed long-term expansion of HSC (Fig. S2). To our surprise, condition D7 also allowed another population of adherent fibroblast-like cells to expand at the same time during HSC expansion (Fig. 1B, Table S1, Table S2). We confirmed this for five independent UCB units. Condition D7 consists of DMEM containing Flt-3 ligand, SCF, MGDF, IL-6 and 10% pooled human AB serum. SCF, Flt-3 and MGDF were added on day 0 and IL-6 was added on day 7 or 10.

The total cell count as well as the number of HSC and colony forming cells (CFC) increased in all 3 UCB units tested (Table S2). In addition, after removing non-adherent cells (HSC) during d14 of culture, we reproducibly observed a population of adherent stromal cells, which appeared to fulfill all criteria of MSC as described below (Fig. 1B). Thus, two different stem cell populations, HSC and MSC, can simultaneously expand from UCB in this novel, stroma-free liquid cell culture (D7) (Fig. 1A, 1C).

At day 28 of culture, adherent spindle-shaped cells were further amplified in MesenCult or in DMEM (Fig. 1B). Fourteen days later, we removed expanded HSC and cultured stromal MSC in MesenCult or in DMEM. Another 14 days later, the cultures that were generated from three independent UCB contained 8′000, 10′000 and 7′300 cells, some with MSC morphology. The cells were passaged for the first time (P1) and were transferred to 25 cm^2^ flasks for expansion. A steady increase in total MSC counts was observed following repeated passages with a higher expansion capacity when a lower number of MSC were plated (80–400 cells/cm^2^). Between P2 and P10, a range of 2.1–800 fold increase in total cell count (mean ± SD: 300±210) was obtained under cultivation with MesenCult, whereas the increase was 6.7–96.4 fold under cultivation with DMEM (51.8±31.4). When seeded at a density of >800 cells/cm^2^, cells expanded 8–92 fold in MesenCult and 6.7–70 fold in DMEM (Table S2). We therefore continued to use MesenCult for *in vitro* expansion of UCB-derived MSC (Fig. 1C). Between P11 and P18, the number of the adherent MSC increased 260–1070 fold (617±206.3) and because of the increased growth rate, cells reached confluency of >80% already by 11 days (Fig. 1D). For this reason, we determined the CFU-F count on day 8 instead of the usual day 11.

Furthermore, we validated condition D7 using HSC (CD34^+^) isolated from 3 independent UCB units. Adherent stromal/MSC were generated in cultures from all selected CD34^+^ samples. These stromal cells were visible by d7 in culture condition D7 and increased extensively by d14 and were thereafter expanded in MesenCult + supplements +5 ng/ml FGF-β (Figs. 1E and 1F). The expansion protocol proved successful in 8 out of 8 independent UCB units tested.

Electron microscopy of cultured cells showed ultrastructural features of MSC. Mitochondria, vacuoles and filaments were present and Weibel-Palade bodies, which are characteristic for endothelial cells, were absent (Fig. 1G). The morphology of UCB-derived MSC appeared to be similar to that of BM-derived MSC [12].

### Phenotypic analysis of MSC and formation of CFU-F colonies

**Figure 2:**
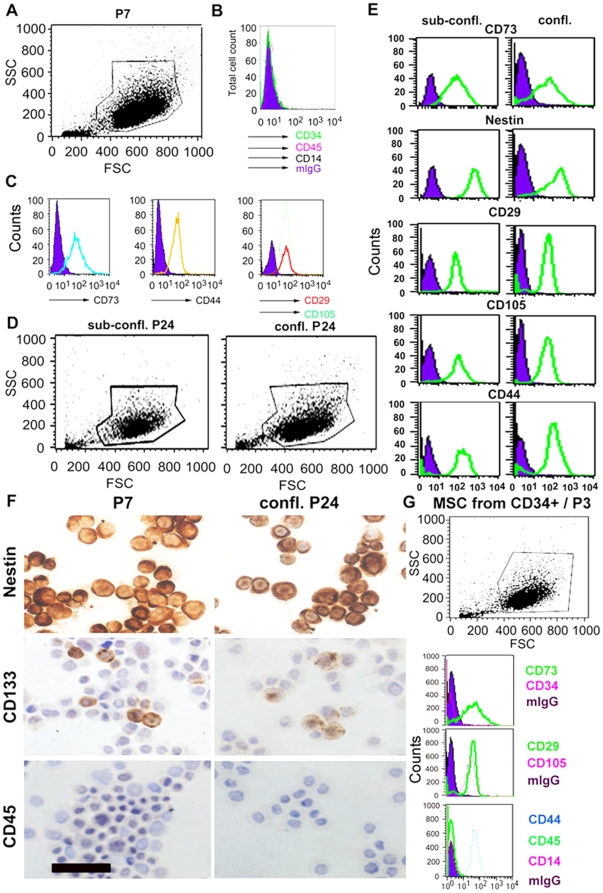
Phenotype of UCB stromal/MSC during long-term culture as analyzed by flow cytometry and immunocytochemistry. Flow cytometric analysis of UCB-derived stromal/MSC cultivated in MesenCult medium with added supplements and 5 ng/ml FGF-β. (A) FSC/SSC plot of MSC at P7. Gate for live cells is indicated. (B) No expression of the haematopoietic markers CD34, CD45 or CD14 on UCB-derived MSC (open histograms). The filled histogram represents the isotype control. (C) Expression of the stromal/MSC markers CD73, CD44, CD29 and CD105 (open histograms). The filled histograms represent isotype controls. (D) FSC/SSC plot of MSC. Sub-confluent vs confluent cultures at P24. Gates for live cells are indicated. (E) Expression of MSC markers (open histograms) at P24 by cells phenotyped at sub-confluent vs. confluent cultures. The filled histograms represent isotype controls. (F) Immunocytochemistry of stromal/MSC prepared from P7 and from confluent culture at P24. Positive peroxidase staining was seen with Nestin and CD133 but not with CD45. (G) Shows cell scatter and phenotype of stromal/MSC obtained from selected CD34^+^ cells at P3. Stromal/MSC were positive for CD73, CD29, CD105 and CD44 (open histograms) and negative for haematopoietic cell markers CD34, CD45 and CD14. The filled histograms represent isotype controls.

We further determined the phenotype of UCB-derived MSC at 80% confluency by flow cytometry analysis. At P3-P4, possible contamination with haematopoietic cells (CD45^+^CD34^+^CD14^+^) was no longer detectable by flow cytometry analysis and 10% of all cells expressed the stem-cell marker CD133. More than 95% of UCB-derived MSC expressed typical MSC proteins CD44, CD29, CD73, CD105 and were also positive for human leukocyte antigen (HLA)-ABC (MHC class I cell surface receptor) but negative for HLA-DR (MHC class II cell surface receptor). This profile remained stable at P4, P7 and P11 (Fig. 2A, B, and C), even as late as passages P13, P18 and P24 (Fig. 2D and E). In addition, the cell population was positive for Nestin (>95%) as determined by flow cytometry analysis (Fig. 2E). The stemness phenotype of UCB stromal/MSC did not change at different confluences [28]. Figure 2D and E display the results of a side-by-side screen of marker profiles of UCB-MSC that were grown in confluent (>95%) vs. sub-confluent (50%) cultures analyzed at passage 24. Immunocytochemistry staining of cytospins prepared from sub-confluent (P7 and P13) and confluent cultures at P24 confirmed the flow cytometry data. Expanded MSC were Nestin positive (range, mean ± SD: 95–98%, 96.3±1.24), CD133^+^ (6–11%, 9.0±2.16), CD45^−^ (Fig. 2F) and mIgG^−^ (data not shown). Stromal/MSC derived from CD34^+^ selected cells had a similar phenotype; cells were negative for haematopoietic markers and positive for MSC markers (Fig. 2G).

**Figure 3:**
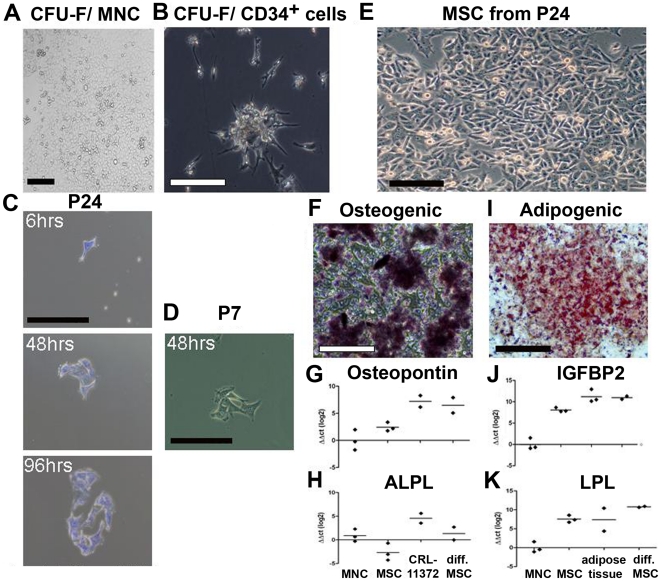
CFU-F colony formation and multilineage differentiation of expanded MSC during long-term culture and following high dilution. (A) Formation of CFU-fibroblast colonies (CFU-F). CFU-F colonies generated from UCB MNC-derived MSC generated large and small CFU-F colonies in MesenCult medium with added supplements and 5 ng/ml FGF-β (scale bar: 150 µm). (B) A CFU-F colony generated from selected CD34^+^ cells derived MSC (d28) (scale bar: 250 µm). (C) Formation of a CFU-F colony following high dilution of outgrowing colonies obtained from confluent culture at P24. One cell adhered after 6 hrs then a small colony formed after 48 hrs and increased in size at 96 hrs stained with Crystal Violet. (D) A CFU-F colony formed 48 hrs after high dilution of outgrowing colonies from sub-confluent culture at P7. (E) A light microscopy of the confluent culture generated at high dilution of stromal/MSC from P24 (scale bar: 150 µm). (F) Osteogenic differentiation of stromal/MSC generated at high dilution stained with alkaline phosphatase at d10 post differentiation (scale bar: 200 µm). (G–H) Real-time PCR for mRNA expression of tissue-specific genes confirms the osteogenic differentiation of UCB-derived MSC as demonstrated by the expression of osteopontin mRNA (G) and ALPL (H). (I) Adipogenic differentiation of stromal/MSC generated at high dilution following Oil red O staining of lipid vacuoles at d21 post differentiation (scale bar: 200 µm). (J–K) Real-time PCR confirms adipogenic differentiation by the expression of Igfbp2 mRNA (J) and LPL mRNA (K). Human cell line CRL-11372 for osteoblasts and human adipose tissue were used as positive controls. UCB-derived MNC and undifferentiated UCB-derived MSC were used as negative controls. All values were normalized to 18S rRNA. Symbols represent individual samples. Horizontal bars depict the average value. ΔΔC_t_ values are depicted in a log 2 scale.

The capacity to form CFU-F colonies is a crucial feature of MSC, and we found that the adherent, MSC-like cells generated large and small CFU-F colonies in MesenCult that was supplemented with 5 ng/ml FGF-β or in DMEM supplemented with 2 mmol/l L-glutamine, 50 IU/ml penicillin, 50 mg/ml streptomycin, 10 ng/ml FGF-β and 20 ng/ml EGF (Fig. 3A). Plating of 1–3×10^5^ MNC/ml after P1 or during HSC expansion resulted in 19–51 CFU-F colonies (mean: 36) in MesenCult and in 28–142 CFU-F colonies (mean: 79.3) in DMEM. With increasing purity of MSC the number of CFU-F increased: we obtained 50–859 (mean ± SD: 372±212.4) CFU-F from a 10^4^ MSC at P2–P10 and 380–788 (mean ± SD: 569 SD ±135) at P11–P18. Also at later passages, we obtained more CFU-F colonies in MesenCult than in DMEM (Table S2). Plating of 50′000–250′000 selected CD34^+^ cells/ml (mean: 136′700) generated 8–19 CFU-F colonies (mean: 12) during HSC expansion (d28) (Fig. 3B). In addition, secondary colonies were formed when outgrowing cells were harvested and replated following high dilution (<10 cells) at early and late passages, P7 and P24, respectively (Fig. 3C and 3D).

### Multilineage differentiation of UCB-derived MSC

**Figure 4:**
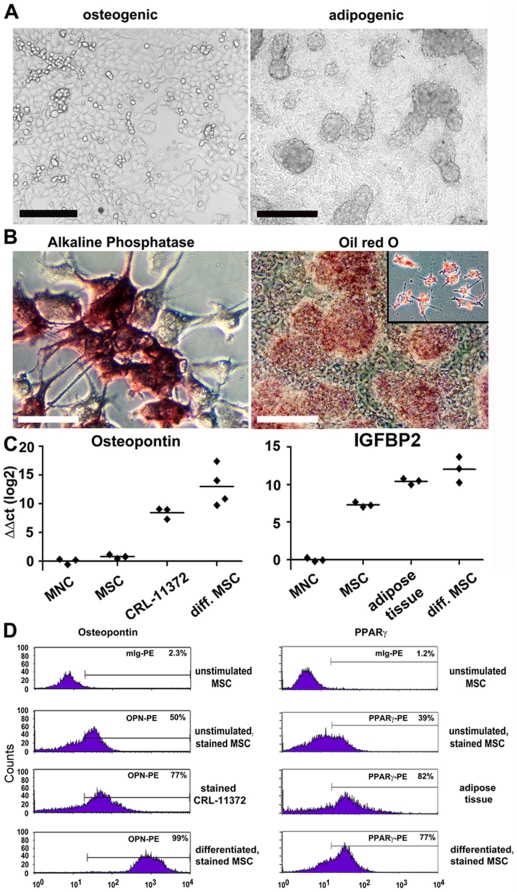
*In vitro* multi-lineage differentiation of UCB-derived MSC into osteoblast- and adipocytes following appropriate induction conditions. (A) Light microscopy of unstained MSC cultures differentiated into osteoblasts (left), adipocytes (right) at 10 and 21 days after differentiation, respectively (scale bar: 200 µm). (B) Osteogenic differentiation was confirmed by alkaline phosphatase activity at d10 post differentiation (left) and adipogenic differentiation by Oil red O staining of lipid vacuoles at d21 post differentiation (right) (scale bar: 200 µm). The inset shows higher magnification of adipocytes stained with Oil red O. (C) Real-time PCR for the mRNA expression of tissue-specific genes confirms the differentiation of UCB-derived MSC. Osteogenic differentiation is demonstrated by the expression of osteopontin mRNA (left) and adipogenic differentiation by the expression of Igfbp2 (right). The osteoblast cell line CRL-11372 and human adipose tissue were used as positive controls. UCB-derived MNC and undifferentiated UCB-derived MSC were used as negative controls. All values were normalized to 18S rRNA. Symbols represent individual samples. Horizontal bars depict the average value. ΔΔC_t_ values are depicted in a log 2 scale. (D) Flow cytometry confirms the protein expression of tissue-specific genes. Osteogenic differentiation was confirmed by staining at day 10 for osteopontin (left) and adipogenic differentiation by staining at d21 for PPARγ (right). Human cell lines (osteoblast: CRL-11372; hepatocyte: Huh7) and human adipose tissue were used as positive controls. UCB MNC and undifferentiated UCB-derived MSC were used as negative controls.

To test whether multilineage differentiation of UCB-derived MSC is possible, we induced differentiation of MSC into osteoblast, adipocyte and hepatocytes (Fig. 3E to K, Fig. 4, Fig. S3, Fig. S4,). Differentiation under osteogenic conditions at P4 and P11 resulted in the generation of spindle shaped cells, which progressively flattened and broadened (day 10 of differentiation is shown in Fig. 4A, left panel). High levels of alkaline phosphatase protein (ALPL) expression and increased cell spreading suggested an osteogenic differentiation (Fig. 4B, left panel). Further, analysis by real-time PCR was in line with flow cytometry analysis and immunocytochemical protein expression data (Fig. 4B and D left panel). MSC cultivated under osteogenic stimuli expressed osteopontin (OPN) and ALPL which are characteristic for osteoblasts (Fig. 4C left panel and Fig. S3A). OPN expression was further confirmed by flow cytometry analysis (Fig. 4D, left panel). Undifferentiated MSC were used as a negative control and did not express significant levels of ALPL or OPN. In contrast, the osteoblast cell line CRL-11372 expressed ALPL and OPN (Fig. 4C left panel and Fig. S3A). When MSC were tested for their potential to differentiate into adipocytes at P4 and P11, morphologic changes in the cells as well as the formation of neutral lipid vacuoles were noticeable as early as day 7 after induction (Fig. 4A, right panel). At day 21, a reduction in nuclear size and accumulation of lipid vacuoles within and around the cells was visualized by staining with Oil red O (Fig. 4B, right panel). Real-time PCR to quantify mRNA expression of genes characteristic for adipocytes revealed that differentiated MSC indeed transcribed adipogenic genes such as Igfbp2, LPL, and PPARγ, similar to a positive control (human adipose tissue) (Fig. 4C, right panel and Fig. S3B and C). We confirmed the expression of PPARγ at protein level using flow cytometry (Fig. 4D, right panel). Importantly, undifferentiated MSC expressed low level of Igfbp2 and LPL but did not express PPARγ. These data confirm that UCB-MSC can differentiate into adipocytes. UCB-derived stromal/MSC passaged in sub-confluent and confluent culture maintained their initial marker profile (see above) and their ability to differentiate as well. At P24, UCB-derived stromal/MSC obtained from confluent culture (Fig. 3E) generated after high dilution could be induced towards osteogenic and adipogenic lineages *in vitro* as demonstrated by ALPL expression (Fig. 3F) and accumulation of lipid vacuoles, respectively (Fig. 3I). Further analysis by real-time PCR confirmed osteogenic (Fig. 3G and H) and adipogenic differentiation (Fig. 3J and K).

### Expression of pluripotency markers in MSC

We have furthermore analyzed the expression of pluripotency markers (e.g. found in embryonic stem cells) such as Nestin, Oct3/4, Nanog, Rex1 and Sox2 in cultures of MSC (Fig. S5A). Nestin was highly expressed in early MSC cultures and its expression remained at high levels until passage 23. Oct3/4 and Nanog showed an increase in the expression over time in sub-confluent MSC cultures, however in confluent cultures both were weakly expressed through all passages.

Rex1 and Sox2 showed strongly increasing expression levels in sub-confluent cultures between P4 and P14, for confluent cultures a slight increase in gene expression was found between P5 and P23. Altogether, we observed increasing expression levels over time for almost all genes tested with a stronger increase seen in sub-confluent cultures compared to confluent cultures, which appear to reach a steady expression level of pluripotency markers around pP17/18.

In addition, we studied the expression of the osteoblast marker osteopontin (Fig. S5B). We found a strong increase in osteopontin expression in sub-confluent MSC cultures between P4 and P14 and a moderate increase of this marker in confluent cultures over time.

### Potential clinical use of UCB-derived MSC

We evaluated the practical implications of the novel methodology developed in the present study. This was conducted in regards to reproducibility, costs, time for generation of MSC, volume of UCB required and the proportion of UCB donations (Fig. S6 and Table S3).

The results show that a small number of MNC or a small volume of UCB can generate an adequate number of stromal/MSC for transplantation of a 70 kg individual (>100×10^6^) (Fig. S5, [29]). Importantly, all cytokines needed to expand MSC from UCB and PB with the culture condition D7 are commercially available under “good manufacturing practice” (GMP) grade.

### Generation of MSC from peripheral blood

**Figure 5:**
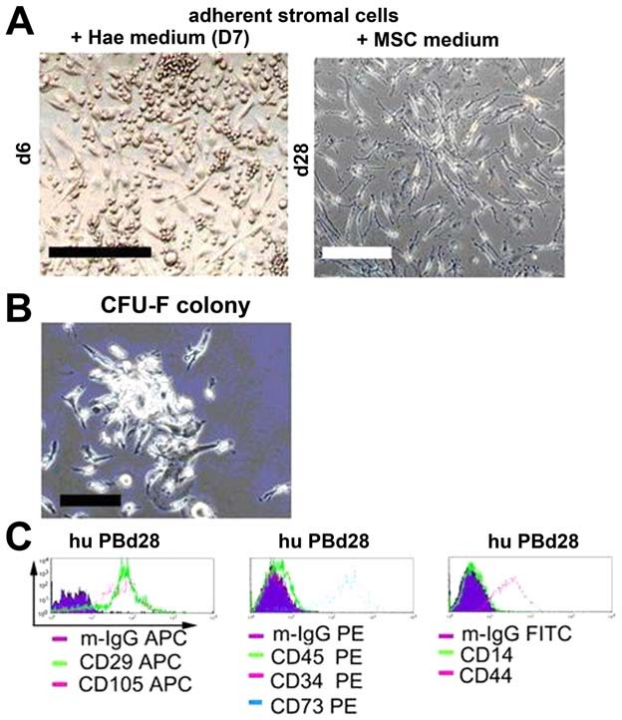
CFU-F colonies of MSC and surface expression of various mesenchymal stem cell markers expressed by MSC generated from steady state PB. Stromal/adherent MSC could be generated from steady state peripheral blood (obtained from 8/8 healthy donors) during the expansion of haematopoietic stem cell in stroma-free liquid culture at day 6 (A, left panel). A confluent growth of stromal adherent cells was obtained at day 28 (A, right panel) (scale bar: 200 µm). (B) A CFU-F colony generated from PB stromal/MSC after P1 (day 28) (scale bar: 100 µm). (C) Flow cytometric analysis of PB-derived stromal/MSC at d28 were positive for stromal/MSC markers CD29, CD105, CD73, CD44 and negative for haematopoietic markers CD45, CD34, CD14. The filled histograms represent isotype controls, respectively.

Further, we tested the expansion potential of our novel culture protocol using other sources than UCB. PB MNC were obtained from 8 healthy volunteers. Unlike the expansion of UCB MSC, MSC derived from PB MNC were expanded only when culture condition D7 was replaced gradually at d14 by MesenCult and not by DMEM. A volume of 250 µl out of the total 1 ml volume of culture condition D7 was replaced twice weekly, by addition of MesenCult and MesenCult supplements plus 5 ng/ml FGF-β until the first passage at d28. None of the culture conditions tested with either DMEM plus different concentrations of serum and cytokines or MesenCult plus MesenCult supplements alone or with > FGF-β at higher concentration (10 ng/ml) allowed the expansion of MSC derived PB MNC (data not shown). Confluent growth of stromal/MSC bearing MSC immature phenotype and the capacity to form CFU-F colonies were obtained from all PB samples (Fig. 5A, B and C).

## Discussion

In recent years, some successful attempts have been made to expand MSC from UCB [4]. However, none of these attempts provided a reproducible protocol to expand MSC, which are rarely found in UCB. In this study, we developed a novel, simple and reliable method which is based on stroma-free liquid culture to expand extensive numbers of multipotent MSC from only a small number of cryopreserved UCB- and PB-derived MNC or CD34^+^ cells. The stroma-free liquid culture referred to as condition D7 maintained the balance between an extensive expansion of HSC and the simultaneous generation of stromal adherent MSC. Therefore, D7 was adopted to establish MSC in all the cultures obtained from UCB or PB. Maintenance of this balance depended on the presence of serum (10% pooled human AB serum), on the combination and the concentration of cytokines (Flt-3, SCF, MGDF, IL-6), the timing of exposure to those and on the culture medium (DMEM) used. Cultivation of MSC in the presence of cytokines and serum found in D7 and in MesenCult did not affect their stemness. Instead, adherent stromal cells were viable and expanded extensively during d7 to d14 of HSC expansion and continued after in MesenCult. In addition to MSC, growth factors and serum maintained long-term expansion of HSC for several months from fetal liver [24] and cord blood ([27]; Peters et al., 2010, unpublished observations). Similarly, cultures of MSC reported using the classical plastic adherent technique always included either serum [17] or serum plus cytokines (such as FGF-β and FGF-α) [9].

In this regard, it is of interest that the use of commercial serum-free media containing low serum (1%) resulted in fewer numbers of stromal cells and/or rapid differentiation of stem cells with lower numbers of HSC and stromal cells (Peters et al., 2010, unpublished observation). The total cell count increased using other culture conditions that were reported previously [25], [26], [27], but the maintenance of HSC (CD34^+^) was only short-term and there was no evidence for concomitant expansion of stromal/MSC. The method developed in the present study to cultivate MNC or CD34^+^ cells in culture condition D7 in stroma-free liquid culture proved very effective. We confirmed the generation of stromal/MSC in 8 out of 8 independent UCB units, 5 MNC-derived and 3 from selected CD34^+^ cells (Table S3). Of note, the success rate for isolating stromal/MSC during HSC expansion in D7 was 100%.

It is also possible that MSC are derived from HSC when selected CD34^+^ cells were cultured in D7. A more homogeneous population of CD34^+^ cells might be obtained by cell sorting to investigate whether those cells generate CD34^−^ cells *in vitro* in D7 culture condition. Previously, we have observed the generation of CD34^−^ cells after 4 to 6 week during long-term culture of fetal liver. Upon flow cytometry analysis these cells appeared as FSC^low^, CD34^−^ and lineage^−^ following the *in vitro* expansion of total cell population (Peters et al., 2010, unpublished observations). Stromal/MSC generated in D7 had a better expansion potential and higher success rate in MesenCult medium (100%) than in DMEM (75%) (Table S3). In MesenCult, expanded cells continued to increase in numbers; showed remarkably little scatter difference in their MSC profiles among samples and among passages; maintained their identity in confluent cultures and their phenotypic profile as well as their differentiation capacity up to the highest passage number we tested in this study, passage 24. In DMEM, the expansion of stromal/MSC after P1 continued in 3 of 4 samples. The expansion potential between P2–P6 was lower in DMEM than MesenCult. After P6, cells increased in size, some detached even when cultures were sub-confluent and cultures needed longer time to reach confluency, >25 days compared to 14 days before P6 (data not shown). In the newly discovered D7 culture condition, a small number of adherent cells, accounting for 5–10% of total expanded cells, were generated every week during UCB expansion. Interestingly, this was also achieved with human PB-derived MNC. Although cultivating PB-derived MNC up to d14 was efficient and reproducible, replacing condition D7 by MesenCult or DMEM for additional 14 days was not. The expansion of stromal adherent cells after d14 decreased and the cultures detached within 48–72 hrs. Unlike UCB, MSC derived from PB MNC were maintained and expanded only when condition D7 was replaced gradually by MesenCult.

It is possible that PB MSC expansion requires the participation of haematopoietic growth factors added as well as the growth factors produced by accompanying haematopoietic cells present in the MNC culture. To maintain their expansion potential PB MNC have to be cultivated in culture condition D7, plus MesenCult and 5 ng/ml FGF-β [30]. In line with these findings, it has been shown that PB-derived MNC and CFU-F differ from BM-derived ones. Both PB-derived MNC as well as PB-derived CFU-F colonies have different growth factors requirements from BM [31], [30].

Adherent cells had the characteristics of stromal cells, the capacity to form CFU-F colonies and were capable of growing into a pure population of MSC when expanded under appropriate culture condition (D7). Besides the generation of CFU-F colonies, genuine MSC must be able to differentiate into different mesenchymal (bone, cartilage, tendon, muscle, adipose tissue, stroma) and possibly into non-mesenchymal tissues (neuronal, endothelial and hepatic) [9], [10], [11] similar to what has been described for MSC from UCB and bone marrow [32], [33].

While exogenous IL-6 was not required for initiating UCB cultures, in particular not for MSC culture, in which IL-6 is secreted by MSC themselves, the addition of IL-6 at 7 to 10 days later was necessary to enhance long-term expansion of both HSC and MSC [34], [35], [36]. The absence of IL-6 after d7 to d10 during culture in D7 had no short-term effect on HSC expansion but was detrimental for the growth, isolation and expansion of MSC (Peters et al., 2010, unpublished observation). In the presence of IL-6, adherent stromal cells increased and formed a confluent cell layer by d14 (Figs. 1B and 1F). Removal of IL-6 for 24–48 hrs from cultures of cord blood and PB resulted in cell death. Cells detached quickly and MSC isolation and/or expansion were no longer possible.

Consistent with these results, a recent study demonstrated that IL-6 inhibited the expansion of white blood cells, but supported the expansion of CD34^+^ cells [34]. We observed by flow cytometry and immunocytochemistry analyses that cultures from early passages (P3 and P7) and late passages (P11, P13, P18 and P24) expressed the CD133 antigen. CD133 has been found to be restricted to stem cells in normal adult tissues [37] and is expressed by non-committed early progenitors of blood cells and endothelial cells [38], nonmalignant neural progenitors, but also by tumor-initiating stem cells in the brain [39]. Recent studies by Tondreau and colleagues showed that selected CD133^+^ cells from human PB give rise to MSC [40]. In our report, cells expressing CD133, which constitute approximately 10% of total cells in culture, were most likely responsible for maintaining the integrity of MSC as shown by the Nestin expression which characterizes the undifferentiated stem cells state [41], [42], the capacity to form colonies in high dilution and the multi-differentiation capacity towards mesodermal lineages. Similarly, CD133^+^ cells might have been responsible for generating stromal/MSC in cultures initiated with selected CD34^+^ cells, in line with published reports where a subpopulation of CD34^+^ cells was characterized by flow cytometry as CD34^+^/CD133^+^ [41], [43], [44]. Further studies will have to indicate whether CD133^+^ cells are the responsible for MSC formation in culture. If this is the case, selection of CD133^+^ could provide an alternative to MSC selection by adherence to tissue culture plastic.

Furthermore, it is highly unlikely that the herewith accomplished generation of stromal/MSC could be a consequence of a possible contamination with MNC (<8–10%) during the selection procedure of CD34^+^ cells (see also Table S3): The contaminant MNC population (4′000 to 25′000 MNC) present during the selection of CD34^+^ cells is very unlikely to form a confluent culture of stromal adherent cells because this number is insufficient to initiate mesenchymal stem cell culture. It was shown previously, that MSC cultures were initiated from 50′000 and 1–3×10^6^ MNC/ml [11], [18] as well was investigated in the herewith presented study: Here we show that at least 1×10^5^–3×10^5^/ml MNC are required to initiate the stromal adherent cell cultures.

Unlike BM-derived MSC, the growth rate of UCB-derived MSC increased with the time in culture indicating a primitive nature [32], [45]. After P11 there was an increase in proliferation rate reaching 205% in total cell count and 153% in CFU-F count. These cells displayed a stable phenotype and retained their potential for differentiation. In the BM, it was reported that senescence of MSC occurred as early as at P11 (47). That was not the case with UCB when MSC were cultured in D7 and thereafter in MesenCult + MesenCult supplements +5 ng/ml FGF-β. The difference is most likely related to the primitive nature of UCB MSC. Alternatively, this might simply be due to the isolation technique which involves the plastic adherence of stromal cells in DMEM + serum. In our hands, senescence of UCB MSC occurred early when MSC were cultured in DMEM + serum ± FGF-β, following 14 days culture in D7.

In the present study, we were able to amplify considerable numbers of multipotent MSC from 1-3×10^5^ MNC in 5 out of 5 independent UCB samples during HSC expansion in stroma-free liquid culture. We observed the presence of those adherent stromal cells in additional 15 out of 15 UCB units during HSC expansion cultures, but we did not process those any further. MSC frequency increased during HSC expansion. This indicates that the combination of elements of early growth factors, serum and medium used to promote the expansion of HSC also promoted the expansion of stromal/MSC. In the present study, no direct comparison was made to evaluate the isolation/cultivation efficiency of UCB MSC under the herewith described culture condition D7 or the classical plastic adherence technique. During the course of establishing MSC from cord blood, we tested 4 independent units of UCB using plastic adherence of MSC. We established adherent MSC only from 1 out of 4 units tested (Peters et al., 2010, unpublished observation), in line with previous reports [3], [8], [21]. In addition to its efficiency and reproducibility, culture condition D7 has additional advantages compared to the classical plastic adherence method: (*i*) culture condition D7 is quicker in initiating stromal/adherent cells; (*ii*) work with culture condition D7 is less laborious, requires a small number of MNC and is less time consuming; (*iii*) culture condition D7 allows many passages and it initiates rare MSC from all UCB and furthermore, from all PB samples.

UCB MSC expressed the pluripotency markers such as Nestin, Sox2, Rex1, Oct3/4 and Nanog analyzed by real-time PCR in confluent and sub-confluent cultures during early, intermediate and late passages up to P23. Gene up-regulation detected for Nestin, Sox2, and Rex1 was the highest in sub-confluent cultures (P5 to P14). Upregulation of Oct3/4 and Nanog reached the maximum level in sub-confluent cultures by P14 but remained weak throughout late and confluent passages. These results are in agreement with the data presented here demonstrating that UCB derived MSC maintained: a) a stable phenotype, b) multidifferential capacity and c) cell stemness despite differences in cell confluency and culture passages. Similarly, cell confluence did not alter the differentiation potential of the osteopointin gene during long-term culture in sub-confluent vs confluent cultures as determined by real-time PCR analysis.

Recently a growing interest in MSC has evolved due to their ease of culture expansion, immunomodulatory activity and differentiation potential. The clinical spectrum of potential therapeutic application includes many diseases such as steroid refractory graft versus host disease, multiple sclerosis, diabetes mellitus, etc. [46], [47]. UCB/MSC have already been used as a potential stem cell therapy in many clinical trials, an ongoing area of development in many clinical and biotechnology institutes worldwide [48], [4], [49], [50], [51]. Despite the clinical need for UCB MSC to treat different diseases, no reproducible method for *in vitro* expansion has been published up to date [52]. The methodology presented here provides the means to isolate the very rare population of MSC from blood. Therefore, we recommend the use of this culture strategy to initiate MSC from various tissues starting with D7 culture condition. D7 promotes the expansion of rare stromal adherent cells and potentially facilitate their successful isolation and expansion, e.g. D7 could be used to isolate MSC from other sources such as fetal MSC from maternal blood during normal and abnormal pregnancy.

We evaluated the practical implications of the novel methodology developed in the present study. We show that at P4, >100×10^6^ MSC could be generated from only 0.5×10^6^ MNC or 23 µl UCB, for transplantation of a 70 kg individual (Fig. S6). The benefits of using this novel technology are (a) to reduce time and costs in preparing MNC, (b) to save a large volume of this valuable source of stem cells (UCB) during MNC preparation and (c) to increase the success rate in generating MSC, which we could show to be 100% in our hands.

We have validated our novel culture protocol using other sources. To our surprise, multipotent MSC were successfully generated from human PB obtained from 8 out of 8 healthy volunteers. This cell population may constitute a unique and sufficiently easy, accessible source of autologous cells with future clinical implications.

## Supporting Information

Figure S1 **A step by step procedure for the generation of MSC during HSC expansion in stroma-free liquid culture.**
(DOC)

Figure S2 **Immuno-peroxidase staining of expanded UCB HSC (CD34^+^) at week 6 during stroma-free liquid culture.** (**A**) Control cells obtained from BD Biosciences, show a mixture of stained CD34^+^ cells (3%) and unstained MNC (97%) (scale bar: 150 µm). (**B**) Expanded MNC showing the increase in CD34^+^ cell population at week 6 in D7 culture condition (scale bar: 150 µm).
(DOC)

Figure S3 **Real-time PCR analysis of MSC differentiated from UBC.** mRNA expression analysis of genes characteristic for particular cell types (e.g. osteoblasts, adipocytes and hepatocytes) was performed with UBC-derived MSC differentiated in various culture conditions (e.g. osteogenic, adipogenic, hepatogenic). (**A**) ALPL mRNA expression for osteoblasts, (**B**) LPL and (**C**) PPAR mRNA expression for adipocytes and (**D**) CK14 and (**E**) CK18 for hepatocytes was performed. All values were normalized to 18S rRNA. Symbols represent individual samples. Horizontal bars depict the average value. ΔΔC_t_ values are shown in a log 2 scale. CRL-11372: Human osteoblast cell line. Huh7: Human hepatoma cell line. Adipose tissue: human adipose tissue.
(DOC)

Figure S4 **Differentiation of UCB-derived stromal/MSC into hepatocyte like cells following appropriate induction condition.** Under hepatogenic culture conditions, MSC developed the typical cuboidal morphology of hepatocyte-like cells within 14 days and further matured by day 28 in the presence of oncostatin M (**A**). Hepatocyte differentiation was further confirmed by immunofluorescence staining for albumin at day 28 (**B**) and by real-time PCR that revealed expression of hepatocyte-specific genes such as albumin, CK14 and CK18 (**C**, and Supplementary **Fig 3D and E**). Further, the expression of CK18 was confirmed by flow cytometry (**D**).
(DOC)

Figure S5 **Expression of pluripotency markers in UCB derived MSC.** (**A**) Real-time PCR for the mRNA expression of pluripotency markers confirms the undifferentiated state of MSC in different passages of sub-confluent and confluent cultures. UCB-derived MNC were used as a negative control. All values were normalized to 18S rRNA. Symbols represent individual samples. Horizontal bars depict the average value. ΔΔC_t_ values are depicted in a log 2 scale. (**B**) Expression of the osteogenic marker osteopontin is found in MSC in different passages of sub-confluent and confluent cultures. UCB-derived MNC were used as a negative control. All values were normalized to 18S rRNA. Symbols represent individual samples. Horizontal bars depict the average value. ΔΔC_t_ values are depicted in a log 2 scale.
(DOC)

Figure S6 **Potential clinical use of UCB-derived stromal/MSC.**
(DOC)

Table S1 **Appropriate culture conditions for expansion of HSC and MSC.**
(DOC)

Table S2 **Long-term expansion of MSC from 3 UCB units tested.**
(DOC)

Table S3 **Percentage success rate obtained for generating stromal/MSC from UCB in D7 culture condition.**
(DOC)
