# Supplementary figures and images for: Efficient Generation of Multipotent Mesenchymal Stem Cells from Umbilical Cord Blood in Stroma-Free Liquid Culture
Source: PLoS One. 2010 Dec 30;5(12):e15689. doi: 10.1371/journal.pone.0015689 (PMC3012708; doi:10.1371/journal.pone.0015689)

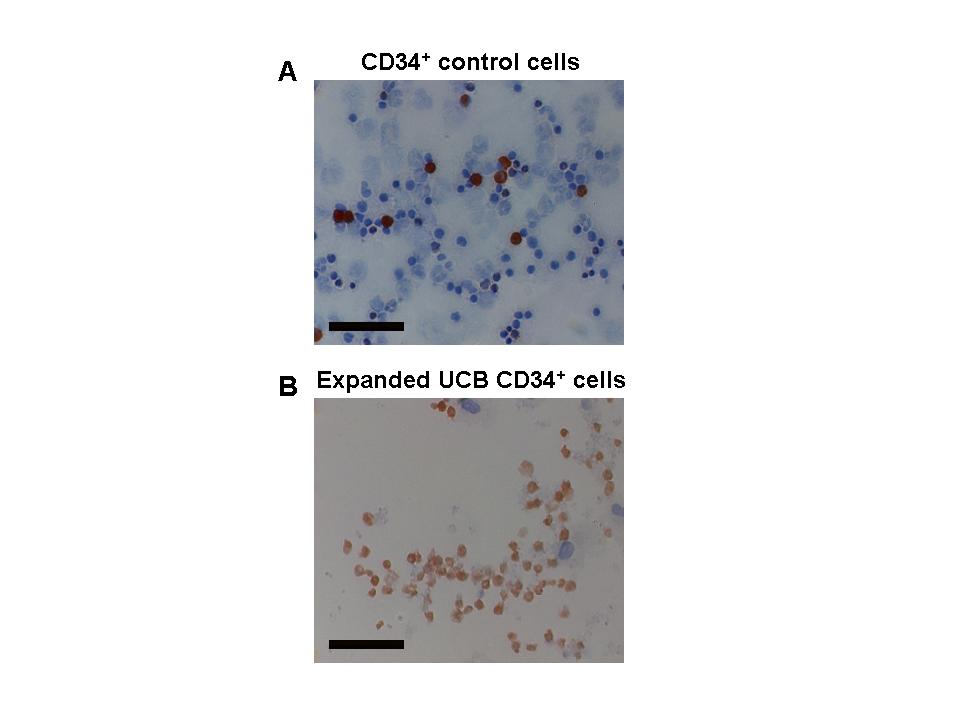

Supplement: Figure S2 — Immuno-peroxidase staining of expanded UCB HSC (CD34+) at week 6 during stroma-free liquid culture. (A) Control cells obtained from BD Biosciences, show a mixture of stained CD34+ cells (3%) and unstained MNC (97%) (scale bar: 150 µm). (B) Expanded MNC showing the increase in CD34+ cell population at week 6 in D7 culture condition (scale bar: 150 µm). (DOC) [file pone.0015689.s002.doc]

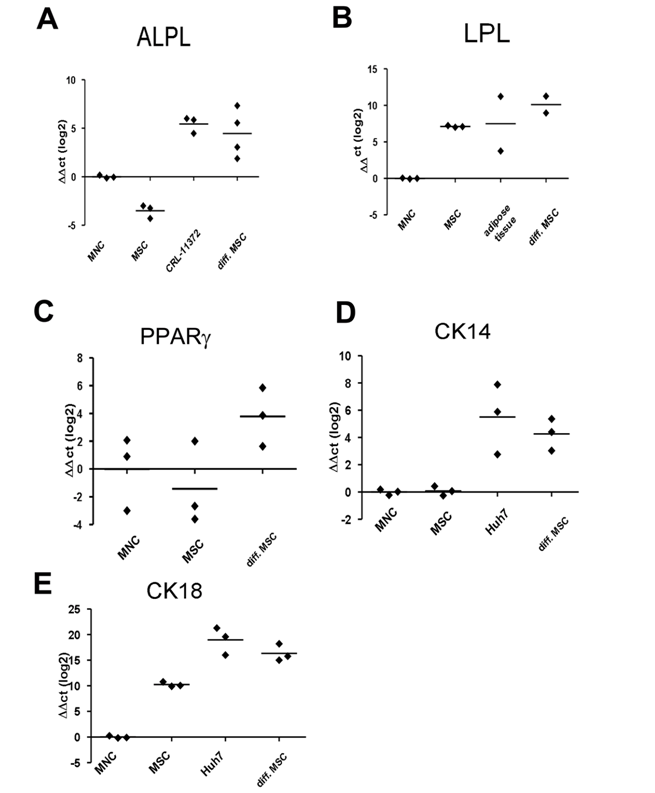

Supplement: Figure S3 — Real-time PCR analysis of MSC differentiated from UBC. mRNA expression analysis of genes characteristic for particular cell types (e.g. osteoblasts, adipocytes and hepatocytes) was performed with UBC-derived MSC differentiated in various culture conditions (e.g. osteogenic, adipogenic, hepatogenic). (A) ALPL mRNA expression for osteoblasts, (B) LPL and (C) PPAR mRNA expression for adipocytes and (D) CK14 and (E) CK18 for hepatocytes was performed. All values were normalized to 18S rRNA. Symbols represent individual samples. Horizontal bars depict the average value. ΔΔCt values are shown in a log 2 scale. CRL-11372: Human osteoblast cell line. Huh7: Human hepatoma cell line. Adipose tissue: human adipose tissue. (DOC) [file pone.0015689.s003.doc]

**
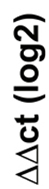

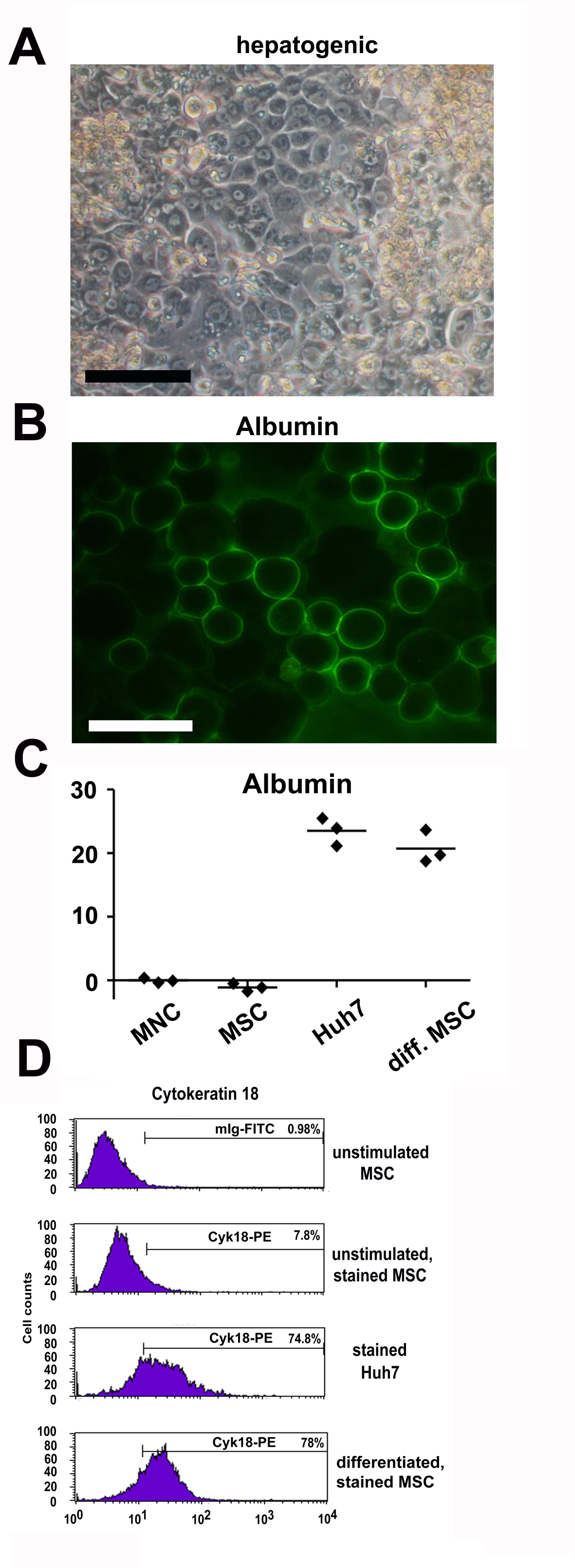
**

Supplement: Figure S4 — Differentiation of UCB-derived stromal/MSC into hepatocyte like cells following appropriate induction condition. Under hepatogenic culture conditions, MSC developed the typical cuboidal morphology of hepatocyte-like cells within 14 days and further matured by day 28 in the presence of oncostatin M (A). Hepatocyte differentiation was further confirmed by immunofluorescence staining for albumin at day 28 (B) and by real-time PCR that revealed expression of hepatocyte-specific genes such as albumin, CK14 and CK18 (C, and Supplementary Fig 3D and E ). Further, the expression of CK18 was confirmed by flow cytometry (D). (DOC) [file pone.0015689.s004.doc]

**
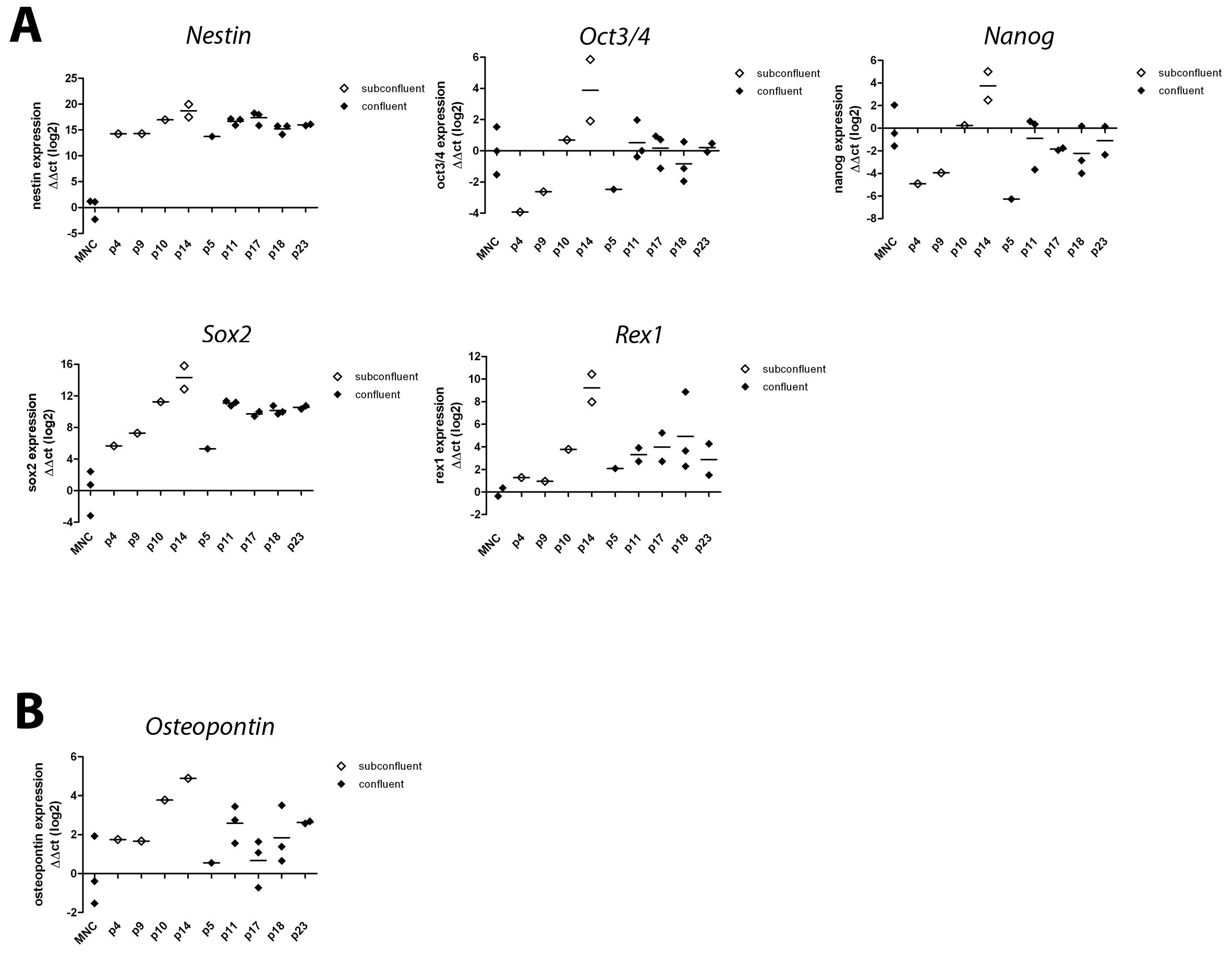
**

Supplement: Figure S5 — Expression of pluripotency markers in UCB derived MSC. (A) Real-time PCR for the mRNA expression of pluripotency markers confirms the undifferentiated state of MSC in different passages of sub-confluent and confluent cultures. UCB-derived MNC were used as a negative control. All values were normalized to 18S rRNA. Symbols represent individual samples. Horizontal bars depict the average value. ΔΔCt values are depicted in a log 2 scale. (B) Expression of the osteogenic marker osteopontin is found in MSC in different passages of sub-confluent and confluent cultures. UCB-derived MNC were used as a negative control. All values were normalized to 18S rRNA. Symbols represent individual samples. Horizontal bars depict the average value. ΔΔCt values are depicted in a log 2 scale. (DOC) [file pone.0015689.s005.doc]
