# Supplementary material for: Efficient Generation of Multipotent Mesenchymal Stem Cells from Umbilical Cord Blood in Stroma-Free Liquid Culture
Source: PLoS One. 2010 Dec 30;5(12):e15689. doi: 10.1371/journal.pone.0015689 (PMC3012708; doi:10.1371/journal.pone.0015689)
Supplement: Table S1 — Appropriate culture conditions for expansion of HSC and MSC. (DOC) [file pone.0015689.s007.doc]

| **Culture name** | **Culture medium + supplements** | **Cytokines** | **Human AB serum** |
| --- | --- | --- | --- |
| Haematopoietic cell expansion culture (D7) used between d0 to d14 of culture | DMEM  + L-glutamine (2 mmol/l)  + penicillin (50 IU/ml)  + streptomycin (50 mg/ml) | Flt-3 (25 ng/ml);  SCF (25 ng/ml); MGDF (10 ng/ml); IL-6 (20 ng/ml). | 10% |
| Mesenchymal stem cell culture (MesenCult) used after d14 of culture | MesenCult +  MesenCult supplements ▲ | FGF-β (5 ng/ml) | / |

▲ Obtained from Stem Cell Technologies, Europe.

d=day.
