# Supplementary material for: Efficient Generation of Multipotent Mesenchymal Stem Cells from Umbilical Cord Blood in Stroma-Free Liquid Culture
Source: PLoS One. 2010 Dec 30;5(12):e15689. doi: 10.1371/journal.pone.0015689 (PMC3012708; doi:10.1371/journal.pone.0015689)
Supplement: Table S2 — Long-term expansion of MSC from 3 UCB units tested. (DOC) [file pone.0015689.s008.doc]

| **Culture Medium** | **Passage**  **No** | **Cell Density**  **MNC/cm^2^** | **Total Cell Count ^a^**  **(range of fold increase)**  **mean ± SD** | **CFU-F Colonies ^b^**  **(range of colony number)**  **mean ± SD** |
| --- | --- | --- | --- | --- |
| **D7** | 0 | 1-3 x 10^5^ | 3.8 - 7 ■  5.63 ± 1.34  19 - 42 ●  28.6 ± 9.74 | / |
| **MesenCult** | 1 | n.d. | n.d. | 19 - 51  36 ± 13.1 |
| **DMEM** | 1 | n.d. | n.d. | 28 - 142  79.3 ± 47.2 |
| **MesenCult** | 2, 3, 6 | >800 | 2.1 - 785  251.7 ± 269.3 | 50 - 618  335 ± 193.5 |
| **DMEM** | 2, 3, 6 | >800 | 6.7 - 94  47.8 ± 30.5 | 58 - 218 *  145 ± 66 |
| **MesenCult** | 2 - 6 | 80 - 400 | 2.1 - 800  275.9 ± 257 | 50 - 639  356.4 ± 190.7 |
| **DMEM** | 2 - 6 | 80 - 400 | 6.7 - 96.4 **  51.8 ± 31.4 | 58 - 269 *  176 ± 78 |
| **MesenCult** | 2 - 10  11 - 18 ^c^ | 80 - 400  80 - 400 | 2.1 - 800  300 ± 210  260 - 1070  617 ± 206.3 | 50 - 859  372.7 ± 212.4  380 - 788  570 ± 135.4 |

^a^ Cells (MNC) were cultivated for 11-14 days, passaged for various times (as indicated) and the total cell count was determined.

^b^ Ten or more MSC attached to each other in the form of a colony (see also Figure 1C) were counted as one colony and total CFU-F colony count was determined between days 8 - 11 after each passage (P).

^c^ Expansion stopped after P 18 and expanded MSC samples were cryopreserved.

■ HSC, total cell count.

● HSC, CD 34^+^ cells.

* Data obtained from one UCB unit.

** Data obtained from two independent UCB units.

n.d. not determined.

At P1 we could not define the MSC cell density or cell count because of high contamination with hematopoietic cells.
