# Supplementary material for: Efficient Generation of Multipotent Mesenchymal Stem Cells from Umbilical Cord Blood in Stroma-Free Liquid Culture
Source: PLoS One. 2010 Dec 30;5(12):e15689. doi: 10.1371/journal.pone.0015689 (PMC3012708; doi:10.1371/journal.pone.0015689)
Supplement: Table S3 — Percentage success rate obtained for generating stromal/MSC from UCB in D7 culture condition. (DOC) [file pone.0015689.s009.doc]

| UCB ▲  No | D7 Culture Condition ■  presence of stromal cells  **d0** **d7** **d14** | Stromal Cells  (d15-d28)  MesenCult Medium  **alone** +**FGF-β** ∆ | Stromal Cells (d15-d28)  DMEM Medium  (FCS)  **10% 15% 20%**  **FGF-β * FGF-β * alone**  **EGF **** | Success  Rate  %  **Mesen- DMEM**  **Cult** | |
| --- | --- | --- | --- | --- | --- |
| UCB 3 MNC | -ve + +++ | + +++ | ++ + + | 100 | 100 |
| UCB 133 MNC | -ve + +++ | + ++ | ++ + n.d. | 100 | 100 |
| UCB 159 MNC | -ve + ++ | + ++ | + **few** **fewer** | 100 | 100 |
| UCB 163 MNC | -ve ++ +++ | ++ ++ | -ve **fewer** **fewer** | 100 | 0 |
| UCB 238 MNC | -ve + + | + |  | 100 |  |
| UCB 237 CD34^+^ | -ve + +++ | ++ |  | 100 |  |
| UCB 220 CD34^+^ | -ve + ++ | +++ |  | 100 |  |
| UCB 228 CD34^+^ | -ve + ++ | ++ |  | 100 |  |

Stromal/MSC counted by microscope /field, (Magnification x40)

**fewer** <5 cells; **few** < 8 cells; **+** = 10-20 cells; **++** = 20-40 cells; **+++** = >40 cells

▲CD34^+^ cell purity =90-92.7%

■ D7 culture condition consists of DMEM containing 10% pooled human AB serum and a

cocktail of cytokines (Flt-3 (25 ng/ml) + SCF (25 ng/ml) + MGDF (10 ng/ml) + IL-6 (20 ng/ml))

were added on day 0 and IL-6 was added on day 7 and 10

∆ FGF-β= 5 ng/ml; * FGF-β =10 ng/ml; ** EGF = 5 ng/ml

n.d. not done; -ve negative
