# Supplementary material for: Efficient Generation of Multipotent Mesenchymal Stem Cells from Umbilical Cord Blood in Stroma-Free Liquid Culture
Source: PLoS One. 2010 Dec 30;5(12):e15689. doi: 10.1371/journal.pone.0015689 (PMC3012708; doi:10.1371/journal.pone.0015689)
Supplement: Supplementary file 5 [file pone.0015689.s001.doc]

Start with MNC or CD34 selected cells

culture 1-3x10^5/ml MNC in 1ml D7 culture condition in 24- well plates

↓

feed wells at d4 with 0.5ml fresh D7

Stromal/adherent fibroblasts like cells become visible by d7

↓

feed cultures again at day 10 with 0.5ml fresh D7

↓

At d14 stop hematopoietic cell expansion, remove D7 medium +hematopoietic cells in suspension

Determine total hematopoietic cell count, CD34 count and hematopoietic colonies

Stromal/adherent fibroblasts + some floating hematopoietic cells remain in the well

↓

At d14 start MSC expansion, add 0.5 ml of non-hematopoietic stem cell growth medium (MesenCult + suppl.+ 5ng/ml FGF-b)

Adherent cells increase rapidly

Colonies of fibroblast –like cells appear in culture

↓

feed cultures again at d21 with 0.5ml fresh MesenCult+ suppl.+ 5ng/mlFGF-b

Count colonies at d28;

Cultures are confluent at d28 (50-80%)

↓

Passage cells in 0.5% trypsin-EDTA

wash once in MesenCult + suppl.,centrifuge at 300g for 10mins

Determine total cell count

↓

Seed 10,000 cells into T25 cell culture flasks

The procedure until P1 takes 28 days
