# Supplementary material for: Efficient Generation of Multipotent Mesenchymal Stem Cells from Umbilical Cord Blood in Stroma-Free Liquid Culture
Source: PLoS One. 2010 Dec 30;5(12):e15689. doi: 10.1371/journal.pone.0015689 (PMC3012708; doi:10.1371/journal.pone.0015689)
Supplement: Supplementary file 6 [file pone.0015689.s006.doc]

1. Practical implications of the method to generate and expand UCB stromal/MSC.

↓

Human MSC have been already approved for use in human transplantation
Obtained 100% success rate in generating stromal/MSC from 8/8 units

2. Volume of UCB is required to yeilds e.g. 100 x10^6cells.

↓

▲ ■Start culture (5x10^5 MNC/1ml well) in D7 (do-d14) → 10,000 stromal/MSC (P1, d28)
13 fold increase, 1.3x10^5stromal/MSC in MesenCult+FGF-b (P2, d42)

↓

Split into 4 T75 culture flasks (30,000/flask)
213 fold increase/ flask → 6.39x10^6 stromal/MSC/flask in MesenCult+FGF-b (P3, d 56) x4

↓

Culture again 30,000 stromal/MSC / T75 flask (x852 flasks)
192 fold increase/ flask → 5.76x10^6 stromal/MSC/flask in MesenCult+FGF-b (P4, d68) x852

3. Proportion of UCB donations will provide this yeild.

↓

MNC were prepared from 4 UCB units after 11-15 hr (13.5±1.5) of collection
Volume → 55-80ml (m=70.5ml ±9.39)
MNC count → 0.25-3x10^9 (m=1.6x10^9±1.16x10^9)

↓

22x10^6 MNC/1ml

↓

23ul of UCB yeilds >100x10^6 Stromal/MSC at P4

▲ A 2ml D7 culture condition medium is required between d0-d14, see Supplemental Fig.1
▲■ The cytokines used in this expansion (SCF,IL-6, Flt-3 ligand, TPO and FGF-b) are available in a GMP-grade (CellGenix Germany).
D7 consists of DMEM containing (Flt-3 Ligand + SCF 25ng/ml), MGDF (10 ng/ml), IL-6 (20 ng/ml) and 10% pooled human AB serum.
The cost of all reagents is minimal because 2mls of total reagents are needed to generate MSC from a 1ml well /one unit of UCB for 14 d in culture.
